# Utility of the Recombinase Driver CX3CR1-ERT2 Rat Strain in Nicotine Self-Administration

**DOI:** 10.3390/cells15161445

**Published:** 2026-08-11

**Authors:** Ashley M. White, Ashley J. Craig, Daryl L. Richie, Christa L. Corley, Percell T. Kendrick, Kathleen R. McNealy, Michael D. Scofield, Cassandra D. Gipson

**Affiliations:** 1 Department of Pharmacology and Nutritional Sciences, University of Kentucky, Lexington, KY 40508, USA; ashleym.white@uky.edu (A.M.W.); ashley.craig@uky.edu (A.J.C.); daryl.richie@outlook.com (D.L.R.); clco305@uky.edu (C.L.C.); ptke222@uky.edu (P.T.K.J.); kathleen.mcnealy@uky.edu (K.R.M.); 2 Department of Anesthesia and Perioperative Medicine, Medical University of South Carolina, Charleston, SC 29425, USA; scofield@musc.edu

**Keywords:** microglia, nicotine, self-administration, behavior, DREADDs

## Abstract

Smoking remains a leading preventable cause of death, and nicotine is the primary substance responsible for maintaining use of tobacco products. Preclinical rodent models have shown that neuroimmune signaling is dysregulated by nicotine self-administration (SA) within the nucleus accumbens core (NAcore). Microglia are the resident brain immune cell and prior studies have shown that they play an important role in nicotine-related behaviors. However, while transgenic mouse lines allow for specific evaluations of microglia to determine their role in neurobiology and behavior, there are fewer tools available for rats as a model species, thus limiting our ability to evaluate specific contributions of microglia to nicotine SA. A transgenic rat expressing Cre under the control of the CX3CR1 promoter bred on a Long–Evans (LE) background was recently developed, and here we show that NAcore microglia can be virally transduced with designer receptors exclusively activated by designer drugs (DREADDs) without neuronal expression. We further show that CX3CR1::ERT2 rats readily self-administer nicotine and display a characteristic extinction curve. Together, these validation studies lay the foundation for future use of this transgenic rat line to evaluate the specific contributions of microglia within the brain reward pathway to the neurobehavioral underpinnings of nicotine addiction.

## 1. Introduction

The immunomodulatory effects of nicotine have been clinically [1] (Sher et al., 1999) and preclinically [2,3,4] documented; however, the impacts of chronic, volitional nicotine use on neuroimmune signaling are a field in its infancy. Microglia are resident brain immune cells that regulate adaptive and innate immune responses [5], regulate synaptic plasticity [6,7,8] and are a part of the neurovascular unit [9]. Recent studies have implicated microglia in nicotine addiction processes, as microglia within the reward pathway and specifically the nucleus accumbens core (NAcore) play a large role in regulating nicotine-related neurobehavior [10,11,12]. However, our ability to specifically manipulate microglia without impacting neurons to determine causal roles of these brain immune cells in nicotine addiction processes remains limited.

The current neuroscience toolbox for evaluating microglia includes viruses, chemicals to deplete them, and transgenic rodent models. Viruses that have been utilized in prior studies include designer receptors exclusively activated by designer drugs (DREADDs), which incorporate adeno-associated viral vectors (AAVs) with promoters that are thought to be microglia-specific (e.g., CD68; ref. [13]), and AAV variants that allow in vitro and in vivo microglial transduction through directed evolution of the AAV capsid protein to increase efficiency of genetic payload into microglia [14]. Notably, the pAAV-CD68-hM4D-mCherry (Gi) and pAAV-CD68-EGFP DREADD utilized in [13] are commercially available but untested in their ability to penetrate microglia within the brain, as that study infused DREADDs intrathecally. The AAV variants that were discovered a few years later to infect microglia were also found to infect neurons [14], thus making it difficult to tease apart microglial versus neuronal contributions to neurobiology and behavior.

As mentioned above, other methodologies to evaluate contributions of microglia to brain function include depletion methodologies with either a Cx3cr1-Dtr rat, which allows for short-term microglia depletion [15], or with colony-stimulating factor–1 receptor (CSF1R) inhibitors (e.g., PLX5622 or PLX3397), which deplete a proportion of microglia as well as other affect peripheral cells [16] when administered in chow. An advantage of this technique is that microglia can repopulate [17], allowing for bidirectional evaluations of microglia to neurobiology and behavioral outcomes. However, microglia depletion is non-specific and can have off-target effects, limiting the cell-type specificity of this approach.

An additional methodology to evaluate microglia includes frequently utilized transgenic mouse strains, which have been used primarily in other fields of research. Transgenic mice, including the recombinase driver CX3CR1-Cre strains [18], have been used to visualize or manipulate microglia in the context of other pathologies [19]. However, transgenic mice have been found to have different baseline nicotine-related behavior rates compared to wild-type controls, limiting the interpretation of data derived from these models [20]. Endogenous CX3CR1 is a microglia-specific marker, and it is a receptor with the endogenous ligand fractalkine [21]. However, there has been some debate regarding specificity of CX3CR1 to microglia, as there are reports that CX3CR1 expression occurs in other cell types depending on the tissue type [22], or co-localization of CX3CR1 with glial fibrillary acidic protein (GFAP; [23]) a marker of astrocyte function [24], although this has not been evaluated within the brain reward pathway to our knowledge. Some studies have also reported that CX3CR1::cre in mice is not specific to microglia, with significant leakage into neurons [25,26,27]. While there are two constitutive and two tamoxifen-inducible CX3CR1::cre mouse lines [28,29,30], there is only one CX3CR1-ERT2 rat line [31]. In this recent publication, the CX3CR1-ERT2 rat line was validated by demonstration of microglial expression of inducible Cre as well as a lack of difference in cognitive task performance between this transgenic strain and wildtype controls. However, no studies to date have utilized this strain to evaluate microglial contributions to addiction neuroscience.

A large majority of studies modeling nicotine addiction utilize self-administration (SA), which allows for evaluations of volitional nicotine use and is most frequently implemented in rat models [32]. Studies also frequently incorporate extinction training following SA to evaluate the ability to learn to withhold the prepotent response to receive the drug and is typically conducted under withdrawal conditions [3]. While some studies incorporate mouse SA to model aspects of nicotine addiction [33,34], use of mice in nicotine SA is limited to a small number of researchers and can present challenges with catheter patency among other issues. While all currently available methodologies to evaluate microglia have limitations, with some noted above, the microglia addiction field has a significant barrier in that there are no validated rat strains for use in SA procedures. Here, we utilized a commercially available CX3CR1-ERT2 transgenic rat strain (LE-Tg(OTTC1005) CX3CR1-Cre-ERT2), which is a tamoxifen-inducible Cre recombinase strain based on a Long–Evans background. The goals of the current study were threefold. Here we determined if this rat strain (1) displays microglia-specific expression of viral vectors within the NAcore without infecting surrounding neurons, (2) can acquire nicotine SA and extinction, and (3) acquires these behaviors similar to CX3CR1-ERT2(**−**) littermates and wildtype Long–Evans rats.

## 2. Materials and Methods

### 2.1. Subjects

Breeder male LE-Tg(OTTC1005) CX3CR1-Cre-ERT2 rats were purchased from the Rat Resource and Research Center (RRRC) and bred in-house with Long–Evans females purchased from Charles River (Wilmington, MA, USA). Rats were housed in a temperature- and humidity-controlled animal facility within the Division of Laboratory Animal Resources (DLAR) at the University of Kentucky. The colony room was on a 12:12 h light:dark reverse light cycle and rats in the breeding colony had ad libitum access to food and water. Rats in nicotine SA were either CX3CR1-ERT2 (+; *N* = 50), CX3CR1-ERT2 (−; *N* = 15), or purchased from Charles River as an outbred strain control (*N* = 35). Male and female rats were 60–90 days old at the beginning of the study and were food restricted to 85% of free feed weight throughout SA experimentation. A total of 16 CX3CR1-ERT2 (+), 2 CX3CR1-ERT2 (−), and 2 wild-type (WT) Long–Evans rats were excluded from the study due to loss of catheter patency. Rats were also removed from the current study due to failure to meet SA criteria (CX3CR1-ERT2 (+), *N* = 4; CX3CR1-ERT2 (−), *N* = 1) or lack of DREADD expression/improper cannula placement (CX3CR1-ERT2 (+), *N* = 3). Finally, rat numbers per group are CX3CR1-ERT2 (+; *N* = 27, M = 11; F = 13), CX3CR1-ERT2 (−; *N* = 12, M = 6; F = 6), or purchased from Charles River as an outbred strain control (*N* = 33, M = 16; F = 17). All animal use practices were approved by the Institutional Animal Care and Use Committee (IACUC) of the University of Kentucky (UK; Protocol #2020-3438).

### 2.2. CX3CR1-ERT2 Genotyping

Ear punches were collected from all animals on post-natal day 21. In separate 1.5 mL microcentrifuge tubes, ear punch tissues were incubated in lysis buffer and Proteinase K (Invitrogen (Carlsbad, CA, USA), ~2 mg/mL, 24 µL total volume) at 55 °C for 30 min, vortexed, and pelleted (18,000× *g*). Samples were incubated for an additional 30 min at 55 °C, vortexed, then resuspended in distilled water up to 200 µL total volume, and pelleted (18,000× *g*). The samples were heated at 100 °C for 10 min, cooled to room temperature, and pelleted at 18,000× *g* for 20 min at 4 °C. Next, 100 µL of the supernatant containing the genomic DNA was transferred to a new 1.5 mL microcentrifuge tube, and standard PCR amplification was used for genotyping.

Subjects were genotyped using the following primers: CX3CR1 F103289 (5′-TCAGGGTGG CCCATAACCAC-3′) and CreETT2 R713 (5′-AGGTAGTTATTCGGATCATCAGCTTAC AC-3′) which produce a 1017 bp amplicon spanning the 5′ end of the CX3CR1-Cre-ERT2. Primers CX3CR1 F103289 (5′-TCAGGGTGGCCCATAACCAC-3′) and CREERT2 R246 (5′-CAGTGA AGCTTCGATGATGGG C-3′), which produce a 550 bp amplicon spanning the 5′ end of the CX3CR1-Cre-ERT2. Primers CreERT2 F1206 (5′-CAGTGAAGCTTCGATGATGGGC-3′) and CX3CR1 R104157–5′–GCCAGATTTCCTGCAGGACCT-3′), which produce a 1553 bp amplicon spanning the 3′ end of the CX3CR1-Cre-ERT2. All rats that underwent infusion of viral constructs were confirmed CX3CR1-ERT2 transgene-positive.

### 2.3. Surgical Procedures

CX3CR1-ERT2 (+) rats included in the immunohistochemistry study underwent surgery for viral infusions targeting the NAcore as previously described [35]. Rats included in the nicotine SA study underwent jugular catheter surgery, and only CX3CR1-ERT2 (+) rats received viral infusion surgery. All rats were anesthetized using ketamine hydrochloride (80–100 mg/kg, i.m.) and xylazine (8 mg/kg, i.m.) and underwent surgical implantation of intravenous jugular catheters as well as stereotaxically infused viral delivery within the NAcore. Intravenous jugular catheters (made from polyurethane tubing; BTPU-040; Instech (Plymouth Meeting, PA, USA)) were inserted 2.5–3 cm into the right jugular vein and were threaded subcutaneously to the posterior side of the animal, where it was connected to an indwelling back port (Instech). Dental cement (Parkell; Edgewood, NY, USA) was used to adhere the catheter to the port. The indwelling port was sutured using 4–0 vicryl braided suture (Ethicons; Cincinnati, OH, USA) subcutaneously ~2 cm caudal from the shoulder blades. Following jugular vein catheterization, animals were immediately transferred to a rat stereotaxic frame and guide cannulae were bilaterally aimed at the NAcore (A/P: 1.5, M/L: 2.0, D/V: −5.5) according to a stereotaxic atlas [36]. Viral vectors encoding DREADDs for microglia infection were infused into the NAcore of CX3CR1-ERT2 animals: pAAV-EF1a-DIO-hM4D(Gi)-mCherry (inhibitory; titer: 1.2 × 10^13^ vg/mL; Addgene, #50461), pAAV-EF1a-DIO-hM3D(Gq)-mCherry (excitatory; titer: 1.3 × 10^13^ vg/mL; Addgene, #50460; packaged into an AAV5 vector by UNC Vector Core), or pAAV5-EF1a-DIO-mCherry (control; i.e., only mCherry-expressing; titer: 1.5 × 10^13^ vg/mL; Addgene, #44362) at a volume of 0.5 μL per hemisphere. Ef1a was selected as the promoter because it is abundantly expressed in all cell types [37], it is double floxed, and both Gq- and Gi-coupled versions are commercially available. Rats were immediately administered cefazolin (100 mg/kg, i.v.) and heparin (10 U/mL, i.v.) and for 7 consecutive days during the recovery period. Meloxicam (1 mg/kg, i.v.) was given immediately and for the first 3 d of the recovery period. For rats in the SA study, heparin (10 U/mL, i.v.) was administered daily. All rats received 4-hydroxytamoxifen (20 mg/kg, I.P.; ref. [38]) 3 weeks following virus infusion and 2 days prior to experimentation.

### 2.4. Food Training Procedures

All rats in the nicotine SA study underwent food training on the sixth day of postoperative care. Food restriction to maintain 85% of free feed weight was implemented a minimum of 2 h before food training initiation and was maintained for the duration of the experiment. Food training sessions were 15 h in duration, and one active lever press resulted in the delivery of one food pellet (fixed-ratio-1 (FR-1), schedule of reinforcement; Bio-Serv, 45 mg/pellet). Concurrently, one food pellet was delivered every 20 min regardless of response. Light and tone stimuli were not paired with pellet administration. Food training criterion was set to 2:1 active to inactive lever presses throughout the session and a minimum of 200 active lever presses. Rats that did not pass the initial food training session completed a second session before beginning SA procedures (N = 3, all CX3CR1-ERT2 (−)). Rats unable to pass food training procedures were excluded from the study (N = 8). All food training, SA, and extinction sessions were conducted in modular operant conditioning chambers (13 ENV-008, 15 ENV-007; Med Associates; Fairfax, VT, USA), which have been previously described in detail [35].

### 2.5. Nicotine SA Procedures

Nicotine infusions (0.06 mg/kg/infusion) were paired with a compound stimulus (light + tone) and were followed by a 20-s timeout period. Nicotine was delivered across 5.9 s at a total volume of 0.1 mL of nicotine per infusion. Infusions were paired with cue lights, located above each lever, and a 2900-Hertz tone was presented throughout the duration of the nicotine infusion. Active lever presses during the timeout period were recorded but did not result in additional nicotine infusions. An inactive lever was extended at all times and responses were recorded; however, presses yielded no programmed consequences or rewards. Session duration was 2 h in length and a FR-1 schedule of reinforcement was used. All animals were required to complete a minimum of 10 sessions before moving into extinction with the following criteria: ≥10 nicotine infusions per session and ≥2:1 active/inactive lever press ratio. Additionally, extinction can be defined as either (1) the lack of delivery of a reinforcer that was previously delivered after a response (e.g., responses on the active lever no longer result in nicotine infusions), or (2) as the absence of a contingency between response and reinforcer (e.g., the nicotine infusion occurs regardless of an animals’ response; see refs. [39,40]). Given that the reinforcer here (the nicotine infusion) was not delivered following a time-out active lever press (these types of responses led to no programmed consequence), the latter definition applies to these types of responses and are therefore distinct from active lever presses that directly result in the initiation of a nicotine infusion. Thus, these two types of lever presses were analyzed separately in the current study.

Following 10 non-consecutive criteria-making sessions, rats were moved to the extinction phase, which took place in the same boxes as self-administration. During the extinction phase, active lever presses no longer resulted in nicotine infusions or associated cues. A minimum of 14 2-h extinction sessions was required for each animal. Extinction criteria were set at <30 active lever presses on the last day of extinction.

### 2.6. Immunohistochemistry

Rats were anesthetized with an overdose of euthasol (0.25 mL/kg i.p.) and transcardially perfused with 0.01 M PBS followed by 4% paraformaldehyde. Brains were then coronally sliced on a vibratome at 60 µm for immunohistochemistry. All antibodies and dilutions are listed in Table 1. Sections containing the NAcore and cannula guide tracks were first incubated in anti-Iba-1 primary antibody that was diluted in 1% BSA, 0.3% Triton, 0.05% azide, and 0.01 M PBS for 24 h at room temperature on a shaker. Sections were then rinsed with 0.01 M PBS three times for 30 min each, then incubated in a secondary antibody conjugated to Alexa Fluor 488 for 2 h. Sections were mounted onto glass slides and coverslipped using an anti-fade mounting medium. Next, to determine if the virus infected microglia that expressed the Cre (+) genotype, NAcore sections were simultaneously stained with 2 primary antibodies (anti-Iba1 and anti-CX3CR1) that were diluted in 1% BSA, 0.3% Triton, 0.05% azide, and 0.01 M PBS for 24 h at room temperature on a shaker. Sections were then rinsed and incubated in secondary antibodies conjugated to Alexa Fluor 488 and Alexa Fluor 594 for 2 h. Sections were mounted onto glass slides and coverslipped using an anti-fade mounting medium containing DAPI to visualize cell bodies. Next to show colocalization of infected microglia without infected neurons, NAcore sections were incubated in 2 primary antibodies (anti-Iba1 and anti-NeuN) that were diluted in 1% BSA, 0.3% Triton, 0.05% azide, and 0.01 M PBS for 24 h at room temperature on a shaker. Sections were then rinsed and incubated in secondary antibodies conjugated to Alexa Fluor 488 and Alexa Fluor 594 for 2 h. All confocal micrographs were captured using a Nikon A1R Inverted Confocal Microscope (Long Beach, CA, USA).

### 2.7. Statistical Analysis

To evaluate if infusion of viral constructs impacts nicotine self-administration prior to actuator delivery, Lever discrimination during food training was compared as a function of virus group and sex using a two-way ANOVA. Lever press data, such as drug infusions and lever press discrimination (active, inactive) across self-administration sessions, were analyzed using linear mixed effects (LME) modeling (JMP Pro 17 software from SAS; Cary, NC, USA). Session was evaluated as a continuous variable, whereas virus and sex were evaluated as between-subjects variables. Subject was treated as a random factor with random intercepts and random slopes. The group × session, sex × session, and group × sex × session interactions were included in each model to determine whether the slope of the session effect varied by virus group and/or sex, and whether consumption/discrimination varied between groups or sexes as sessions progressed. The control virus group was the reference group in all analyses unless otherwise specified for post hoc testing. Tukey’s HSD was used as post hoc pairwise comparisons on nominal variables where appropriate (α = 0.05). Lever discrimination was assessed as the percentage of active/active + inactive lever presses (including infusion-earning and timeout active lever presses, as well as all inactive lever presses).

A separate cohort was used to compare CX3CR1-ERT2 (+) (collapsed across virus type) rats to CX3CR1-ERT2 (−) littermates and wild-type Long–Evans rats. Similar to the first behavioral experiment, lever discrimination during food training was compared as a function of virus group and sex using a two-way ANOVA. Lever press data such as drug infusions and lever press discrimination (active, inactive) across self-administration sessions were analyzed using linear mixed effects (LME) modeling (JMP software from SAS). Session was evaluated as a continuous variable, whereas genotype and sex were evaluated as between-subjects variables, with the genotype × session, sex × session, and genotype × sex × session interactions included in each model. Total nicotine consumption summed across the 10-day SA experiment was compared as a function of genotype and sex using a two-way ANOVA.

## 3. Results

### 3.1. Cre-Dependent Viruses Expressed Within NAcore Microglia

**Figure 1:**
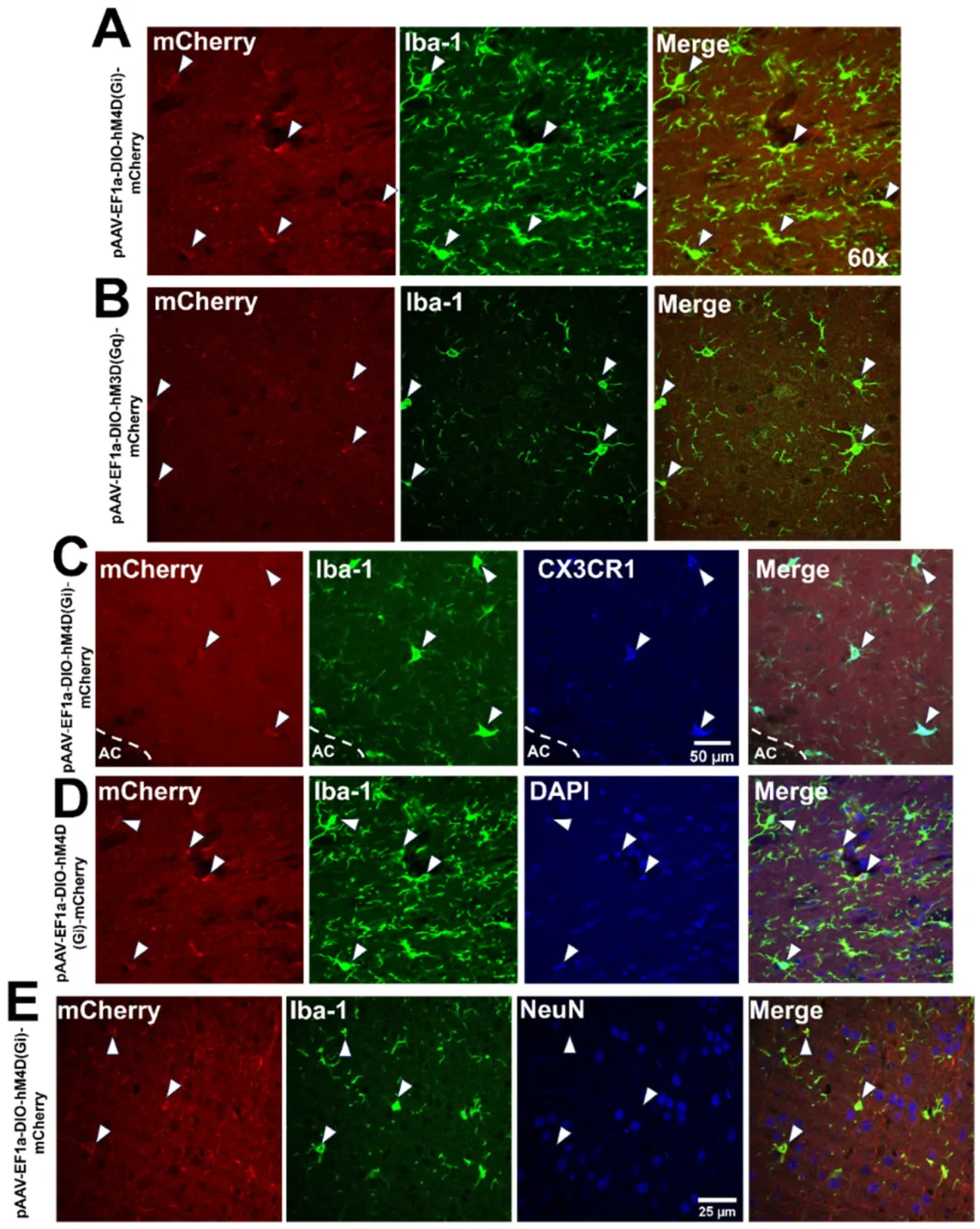
**NAcore microglia but not neurons are transduced with AAV constructs.** Representative immunohistochemical images from animals receiving viral infusions are shown. Slices were stained with either whole-cell microglial marker, Iba1; Cre-recombinase marker, mCherry; microglial receptor, CX3CR1; cell soma marker, DAPI, and/or neuronal cell marker, NeuN. Merged images show overlap of microglial and viral markers, indicating infection of microglia with viral constructs without neuronal infection. AC = anterior commissure, denoted by a dotted line. White arrows = cells displaying colocalization. Scale bars in (**A**–**C**) (large images) = 50 µm; scale bars in (**D**,**E**) (large images) = 25 µm. Scale bars for insets in (**A**–**C**) = 3 µm; scale bars for insets in (**D**,**E**) = 2 µm.

Immunohistochemistry was first conducted to determine if the fluorescent mCherry tag from the viruses was expressed in NAcore microglia following intracranial viral delivery. Images were taken using a Nikon A1R Confocal Microscope. Figure 1A shows that mCherry from the Gi viral construct was successfully observed within NAcore Iba1(+) cells. Similarly, we found co-localization of mCherry with NAcore Iba1(+) cells with the Gq construct (Figure 1B). To determine specificity to CX3CR1-expressing cells, we next evaluated co-localization of mCherry from the Gi construct with CX3CR1 and Iba-1. Notably, cells that were mCherry(+) expressed both microglia markers (Figure 1C), demonstrating that the CX3CR1-ERT2 transgenic rat strain allowed for AAV infection of microglia. Finally, we confirmed somatic expression of mCherry with co-localization of the fluorescent tag with DAPI, which visualizes nuclear DNA (ref. [41]; Figure 1D). Next, we determined if mCherry from the Gi construct co-localized with the neuronal marker NeuN. As shown in Figure 1E, there was no co-localization of mCherry with NeuN, demonstrating specificity of mCherry to non-neuronal cells within the NAcore.

### 3.2. CX3CR1-ERT2 (+) Rats Acquire Nicotine SA and Extinction

**Figure 2:**
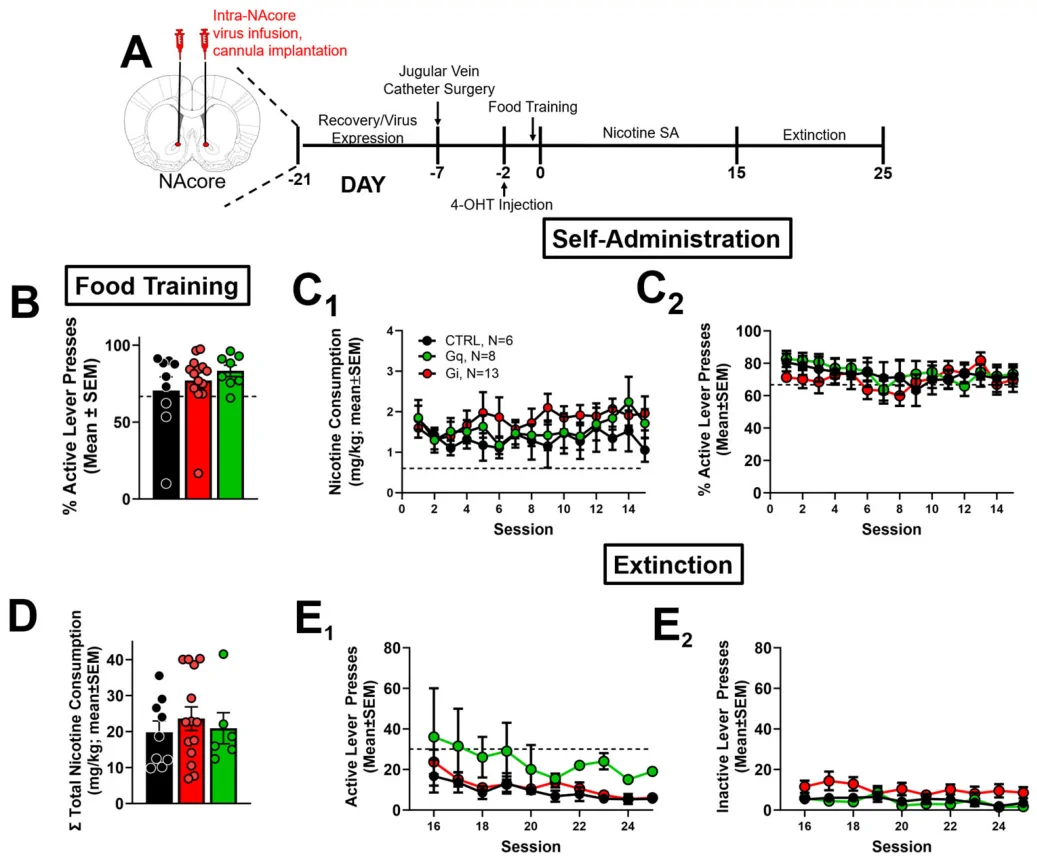
CX3CR1-ERT2 rats with viral constructs acquire nicotine SA and extinction. (**A**) Experimental timeline for CX3CR1-ERT2 rats (G_q_ = 8; M = 5, F = 3 (green), G_i_ = 13; M = 6, F = 7 (red), CTRL = 6; M = 2, F = 4, black). (**B**) Food training where animals met a 2:1 active-to-inactive lever pressing ratio compared across virus groups with no significant differences (*p* > 0.05). (**C_1_**,**C_2_**) All CX3CR1-ERT2 rats acquired lever responding to the active lever to receive IV infusions of nicotine (0.06 mg/kg/infusion) and no statistically significant differences were detected in lever discrimination regardless of virus. Dotted line in (**C_1_**) = infusion acquisition criteria (10 infusions or 0.6 mg/kg); dotted line in (**C_2_**) = lever discrimination criterion of 66.67% response allocation to the active lever. (**D**) CX3CR1-ERT2 rats regardless of virus had no significant differences in total nicotine consumption across the 15-day timeline. (**E_1_**) All CX3CR1-ERT2 rats regardless of virus showed a reduction in active lever pressing behavior during extinction training to <30 lever presses per session (indicated by the dotted line). (**E_2_**) Rats show no genotype-specific differences in inactive lever pressing behavior during extinction training.

First LME was conducted on lever discrimination during food training as a function of virus and sex. No main effect of sex (F1,52 = 0.65, *p* = 0.424) or virus (F1,52 = 0.21, *p* = 0.890) was found, indicating that all rats acquired food training. Baseline nicotine consumption was compared during nicotine SA acquisition as a function of virus groups following viral administration (see Figure 2A for an experimental timeline). LME revealed no significant main effects of sex (F1,25.838 = 2.216, *p* = 0.149), group (F2,17.844 = 0.153, *p* = 0.859), or session (F1,17.30 = 1.296, *p* = 0.271) on nicotine consumption. There were also no sex × group (F2,26.47 = 1.075, *p* = 0.356), sex × session (F1,20.590 = 0.304, *p* = 0.587), or sex × group × session (F2,20.158 = 0.101, *p* = 0.905) interactions (Figure 2B). LME also revealed no significant main effects of sex (F1,25.838 = 2.216, *p* = 0.149), group (F2,19.080 = 0.138, *p* = 0.275), or session (F1,18.334 = 1.009, *p* = 0.328) on discrimination. There were also no sex × group (F2,27.941 = 1.278, *p* = 0.7596), sex × session (F1,18.682 = 0.353, *p* = 0.560), or sex × group × session (F2,18.586 = 0.271, *p* = 0.766) interactions (Figure 2(C_1_,C_2_)). These results indicate that virus administration did not change nicotine SA acquisition rates or discrimination regardless of the construct type before tamoxifen administration. When nicotine consumption was summed across experimentation, there were no group F2,18 = 1.10, *p* > 0.356) or sex (F2,18 = 0.30, *p* = 0.593) differences in the total amount of nicotine consumption across the 15-day SA experiment (Figure 2D). All CX3CR1-ERT2(+) rats regardless of virus showed a reduction in active lever pressing behavior during extinction training to <30 lever presses per session (Figure 2(E_1_,E_2_)).

### 3.3. CX3CR1-ERT2(+) Rats Acquire Nicotine SA Similarly to CX3CR1-ERT2(−) Littermates and Wild-Type Rats

**Figure 3:**
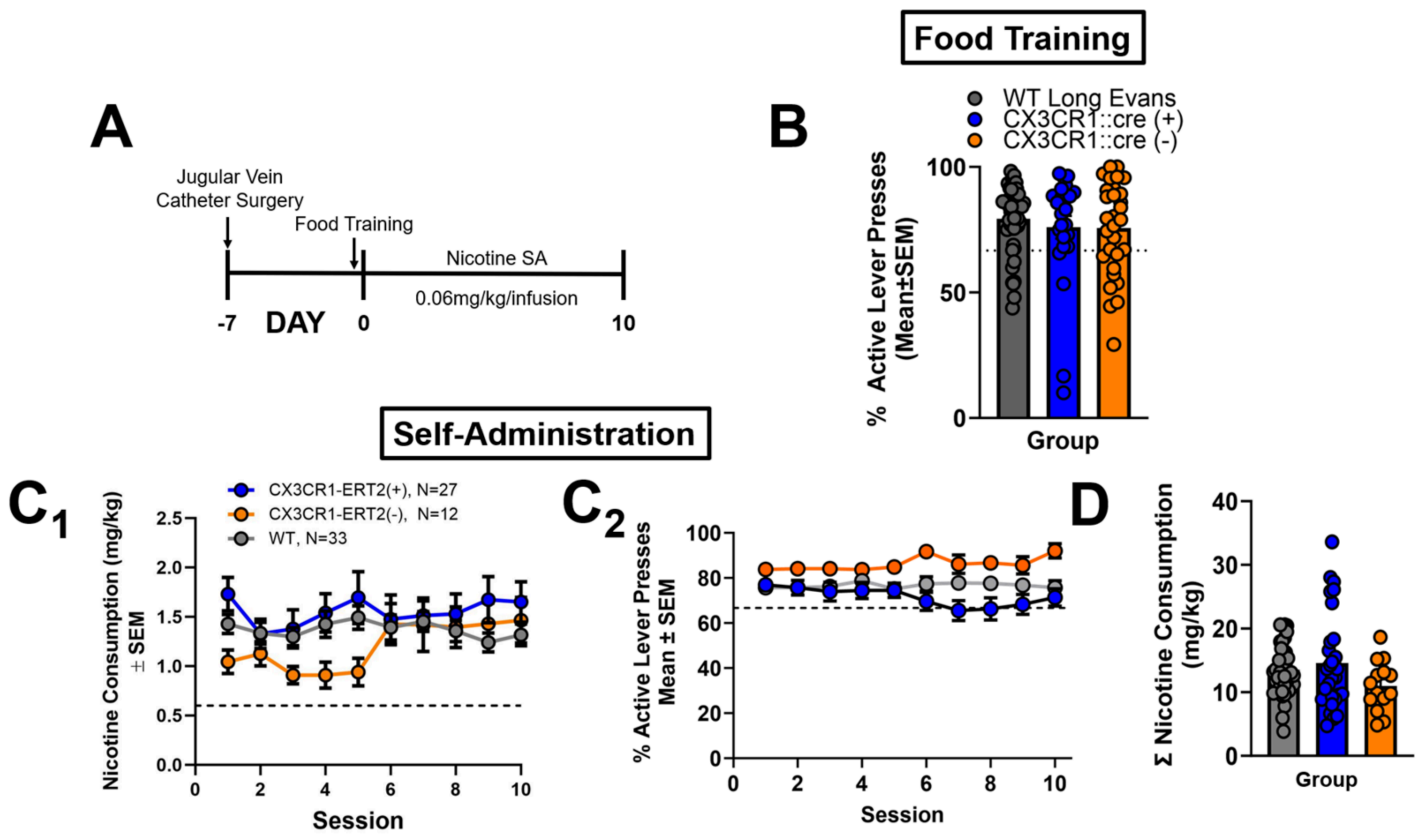
**CX3CR1-ERT2 rats acquire nicotine SA similarly to Cre-negative littermates and wild-type rats**. (**A**) Experimental timeline for comparison across genotype (CX3CR1-ERT2(+) = 27; M = 13, F = 14 (blue), CX3CR1-ERT2(−) = 12; M = 6, F = 6 (orange), WT = 33; M = 16; F = 17(gray)). (**B**) Food training where animals met a 2:1 active-to-inactive lever pressing ratio compared across genotype groups with no statistically significant differences (*p* > 0.05). (**C_1_**,**C_2_**) CX3CR1-ERT2(+) rats acquired lever responding to the active lever to receive IV infusions of nicotine (0.06 mg/kg/infusion) compared to CX3CR1-ERT2(−) littermates and WT-ordered animals. All rats met the discrimination criteria by the end of experimentation, but CX3CR1-ERT2(−) littermates showed a significantly higher lever discrimination as compared to CX3CR1-ERT2(+) (*p* < 0.05). Dotted line in (**C_1_**) = infusion acquisition criteria (10 infusions or 0.6 mg/kg); dotted line in (**C_2_**) = lever discrimination criterion of 66.67% response allocation to the active lever. (**D**) Rats had no significant differences in total nicotine consumption across the 10-day experiment, regardless of genotype.

Data from Figure 2 were collapsed across virus type and compared to CX3CR1-ERT2 transgene (−) littermates, as well as purchased Long–Evans rats. First LME was conducted on lever discrimination during food training as a function of genotype. No main effect of sex (F1,68 = 0.59, *p* > 0.05), genotype (F1,68 = 0.44, *p* > 0.05), or sex × genotype interaction (F1,68 = 0.89, *p* > 0.05) was found, indicating that all rats acquired food training with no statistically significant genotype or sex differences in this effect (Figure 3B). Nicotine consumption across sessions was compared during nicotine SA acquisition as a function of genotype groups (Figure 3(C_1_)). LME revealed a significant main effect of group (F2, 63 = 3.421, *p* = 0.039), but a post hoc pairwise comparison revealed no significant differences between genotypes (*p*s ≥ 0.222). LME revealed no significant main effects of sex (F1, 63 = 0.0237, *p* = 0.878) or session (F1, 63 = 3.31, *p* = 0.076). There were also no sex × group (F2, 63 = 0.019, *p* = 0.981), sex × session (F1, 63 = 0.171, *p* = 0.681), or sex × genotype × session (F2, 63 = 0.083, *p* = 0.920) interactions (Figure 3(C_1_)). These results indicate that the genotype did not change nicotine SA acquisition rates regardless of the sex of the rat. LME was conducted on lever discrimination during nicotine acquisition as a function of sex, genotype, and session. No main effect of genotype (F1,61.807 = 2.062, *p* = 0.136), session (F1,61.379 = 0.029, *p* = 0.866), or sex (F1,66.49 = 0.475, *p* = 0.493). There were also no sex × genotype (F2, 68.314 = 1.063, *p* = 0.351), sex × session (F1, 63.046 = 0.002, *p* = 0.963), or sex × genotype × session (F2, 63.576 = 1.063, *p* = 0.352) interactions. When nicotine consumption was summed across experimentation, there were no sex (F1,73 = 1.53, *p* = 0.220) or genotype (F2,73 = 2.03, *p* > 0.139) differences in the total amount of nicotine consumption across the 10-day SA experiment (Figure 3D).

## 4. Discussion

Here we show that the CX3CR1-ERT2 transgenic rat strain can be used to specifically transduce DREADDs in microglia within the NAcore, and these rats can acquire intravenous nicotine SA and also undergo successful nicotine extinction training. We compared acquisition curves across virus type and across strain and found statistically significant differences in either analysis. As noted previously, evaluations of microglia in addiction neurobiology have been limited due to the tools available to study this cell type in rat models. Our current data reported here support the use of this transgenic rat strain in the addiction neuroscience field to evaluate microglia contributions to substance use, and particularly, in the nicotine addiction field. The CX3CR1-ERT2 rats have previously been shown to exhibit cue-conditioned learning that is not different compared to wild-type controls [31]. Here, we show that these rats exhibit not only cue-conditioned learning for nicotine responding, but also extinction. Together, these findings demonstrate that these rats can adapt their behavior in a context-dependent fashion [42]. Importantly, we did not find significant differences in SA behavior frequently evaluated within the preclinical addiction neuroscience field between CX3CR1-ERT2 (+), CX3CR1-ERT2 (−), and wild-type Long–Evans rat strains. This is of particular importance because prior studies utilizing Cre recombinase driver mouse lines have found baseline differences in nicotine-related behaviors as well as altered baseline profiles of the system being genetically modified (in the prior case, the cholinergic system in ChAT-Cre mouse lines; [20]).

While the current study demonstrates the utility of this rat strain in the ability to infect microglia without neuronal expression of viral constructs as well as SA and extinction behaviors, there are notable limitations that must be outlined. First, it is important to note that the current study did not functionally evaluate the impacts of chemogenetic activation or inhibition on NAcore microglia, including evaluations of transcriptomic, epigenetic, metabolomic, or proteomic changes induced by activation of the DREADD receptors. This is important because the microglia field has taken a complex multidimensional approach to microglial states [5], and chemogenetic activation or inhibition of microglia may have varied impacts on these aspects of microglial reactivity. Indeed, it is unknown how muscarinic DREADD receptors expressed on CX3CR1(+) microglia may shift these dynamic aspects of these immune cells. One study did find that activation of Gi DREADDs expressed in microglia of CX3CR1creER/+:R26LSL-hM4Di/+ transgenic mice inhibited chronic pain and spinal nerve transection-induced microglial reactivity [43]. This study also found that the Gi DREADD reduced interferon regulatory factor 8 (IRF8) and interleukin (IL)-1β expression in Iba1(+) cells, although this study did not confirm that all microglia were DREADD(+). Thus, future studies are needed to determine how chemogenetic modulation of microglia impacts their reactivity profiles.

While our current study demonstrates that this rat strain allows for infection of CX3CR1(+) microglia, this methodology requires these cells to be virally infected with the construct. Here we did not find that all microglia within the NAcore were positive for mCherry (evidenced in Figure 1), indicating that either microglia in the visual field were outside of the area of viral delivery, or that some microglia within the area of viral delivery were not infected with the constructs. This could be due to (1) too little infused volume of the virus to perfuse through the visualized area of the NAcore, (2) some microglia did not express Cre, or (3) some microglia were able to mount an immune response to the viral constructs. The recent report indicating that this rat strain demonstrates inducible Cre in microglia also used an anti-mCherry antibody for increased ability to detect reporter expression [31], and thus it is also possible that microglia within the visualized area were infected with DREADDs but demonstrated a low or undetectable level of mCherry fluorescence. This study [31] also reported a small number of mCherry(−)/Iba1(+) cells, which is in line with our findings here and indicates that this strain may not allow for 100% microglial expression in the area of interest. It is also important to note that this rat strain requires tamoxifen injections. Given that tamoxifen is a selective estrogen receptor modulator and may have sex-specific antiestrogenic effects [44] that could interfere with experimental outcomes, future studies incorporating CX3CR1-ERT2 rats should take this into account in experimental timelines. Here, rats were administered tamoxifen during the early part of nicotine SA acquisition, which supports that tamoxifen induction of Cre can be done post-drug SA acquisition, which can be leveraged in future studies. Importantly, tamoxifen has a half-life of 10.3 h in rats [45], and five elimination half-lives are required for approximately 97% of the bioavailable dose of a drug to be eliminated from the body (here, 51.5 h would be required; [46]). Given this and that no sex differences were found in the nicotine SA acquisition curves, it is unlikely that tamoxifen impacted the measured behavioral outcomes here. As well, another study administered 4-OHT immediately prior to cocaine conditioning and observed measurable cocaine place preference in mice [38]. 4-OHT has been shown to Finally, it is not clear if the CX3CR1-ERT2 rat line has altered constitutive CX3CL1/CX3CR1 signaling, which is important in neuron/microglia crosstalk in brain injury and inflammation [47]. Despite these caveats, this rat strain may allow for more granular in vivo evaluations of microglia to glutamate dyshomeostasis induced by addictive drugs [48], including nicotine [49], as well as cell type-specific dissections of glial contributions to the neurobehavior of nicotine addiction.

Importantly, a practical consideration for implementing this transgenic line is the utility of the CX3CR1-Cre-ERT2-/- littermates. In line with the Three R’s of animal research–Replacement, Reduction, and Refinement [50], we tested these littermates and compared them to wildtype Long–Evans rats to determine if they showed differences in behaviors commonly utilized in preclinical addiction studies. Here we show that for nicotine-related behaviors, the CX3CR1-Cre-ERT2 (−) littermates can be utilized to maximize the use of animals bred for experimentation as well as refine colony management practices. This consideration is important for investigators to maximize the scientific potential for a budget in a given fiscal year since the cost and workload of maintaining a breeding colony is usually more burdensome compared to ordering animals from a commercial supplier [51].

## 5. Conclusions

Here, we show that the inducible recombinase driver CX3CR1-ERT2 rat strain can be successfully used in a model of nicotine SA. Indeed, we show that CX3CR1-ERT2 rats readily self-administer nicotine without significant differences as compared to wildtype and CX3CR1-ERT2 (−) littermate controls, and the viral constructs were successfully expressed within microglia without neuronal co-infection within the NAcore. The use of this transgenic rat model and viral constructs will provide researchers with the toolbox to functionally evaluate microglial contributions to addiction with cell-type and brain region specificity in the future. Taken together, our current study, as well as the prior report establishing this rat strain [31], lays a foundation upon which future studies can identify microglial contributions to addiction neurobiology and behavior.

## Figures and Tables

**Table 1:** Antibodies for immunohistochemistry.

Target	Company	Catalogue Number	RRID	Dilution
Mouse Anti-NeuN antibody	Abcam (Waltham, MA, USA)	ab104224	RRID:AB_10711040	1:500
AlexaFluor 488 goat anti-chicken IgY	Abcam (Waltham, MA, USA)	ab150173	RRID:AB_2636995	1:500
Anti Iba1, Rabbit	Fujifilm (Valhalla, NY, USA)	019-19741	RRID:AB_839504	1:500
Anti CX3CR1, Mouse	Abcam (Waltham, MA, USA)	ab167571	RRID:AB_2916288	1:500
DAPI antibody	Thermo Fisher (Waltham, MA, USA)	D1306	RRID:AB_2629482	1:1000

## Data Availability

The original data presented in the study are openly available in Harvard Dataverse at https://doi.org/10.7910/DVN/NMEBTZ.

## Abbreviations

The following abbreviations are used in this manuscript:

SA	Self-Administration
NAcore	Nucleus Accumbens Core
LE	Long–Evans
DREADDs	Designer Receptors Exclusively Activated by Designer Drugs
AAV	Adeno-Associated Viral
CSF1R	Colony-Stimulating Factor–1 Receptor
GFAP	Glial Fibrillary Acidic Protein
RRRC	Rat Resource and Research Center
DLAR	Division of Laboratory Animal Resources
IACUC	Institutional Animal Care and Use Committee
LME	Linear Mixed Effects
IRF8	Interferon Regulatory Factor 8
IL	Interleukin
